# Multiploid CD61+ Cells Are the Pre-Dominant Cell Lineage Infected during Acute Dengue Virus Infection in Bone Marrow

**DOI:** 10.1371/journal.pone.0052902

**Published:** 2012-12-27

**Authors:** Kristina B. Clark, Sansanee Noisakran, Nattawat Onlamoon, Hui-Mien Hsiao, John Roback, Francois Villinger, Aftab A. Ansari, Guey Chuen Perng

**Affiliations:** 1 Department of Pathology and Laboratory Medicine & the Emory Vaccine Center, Emory University School of Medicine, Atlanta, Georgia, United States of America; 2 Medical Biotechnology Research Unit, National Center for Genetic Engineering and Biotechnology, National Science and Technology Development Agency, Bangkok, Thailand; 3 Office for Research and Development, Faculty of Medicine Siriraj Hospital, Mahidol University, Bangkok, Thailand; 4 Center for Emory Bone Marrow Transplant Center, Emory University, Atlanta, Georgia, United States of America; 5 Division of Pathology, Yerkes National Primate Research Center, Emory University, Atlanta, Georgia, United States of America; 6 Center of Infectious Disease and Signaling Research, National Cheng Kung University, Tainan, Taiwan; UC Irvine Medical Center, United States of America

## Abstract

Depression of the peripheral blood platelet count during acute infection is a hallmark of dengue. This thrombocytopenia has been attributed, in part, to an insufficient level of platelet production by megakaryocytes that reside in the bone marrow (BM). Interestingly, it was observed that dengue patients experience BM suppression at the onset of fever. However, few studies focus on the interaction between dengue virus (DENV) and megakaryocytes and how this interaction can lead to a reduction in platelets. In the studies reported herein, BM cells from normal healthy rhesus monkeys (RM) and humans were utilized to identify the cell lineage(s) that were capable of supporting virus infection and replication. A number of techniques were employed in efforts to address this issue. These included the use of viral RNA quantification, nonstructural protein and infectivity assays, phenotypic studies utilizing immunohistochemical staining, anti-differentiation DEAB treatment, and electron microscopy. Cumulative results from these studies revealed that cells in the BM were indeed highly permissive for DENV infection, with human BM having higher levels of viral production compared to RM. DENV-like particles were predominantly observed in multi-nucleated cells that expressed CD61+. These data suggest that megakaryocytes are likely the predominant cell type infected by DENV in BM, which provides one explanation for the thrombocytopenia and the dysfunctional platelets characteristic of dengue virus infection.

## Introduction

Bone marrow (BM) is the principal site for blood cell formation; the daily production of which in adults is 2.5 billion red cells and platelets each, and 1.0 billion granulocytes per kilogram of body weight. The bone marrow compartment is a highly dynamic environment; even small changes can lead to a very significant modification in the cellular constituents in the corresponding peripheral blood.

There is extensive evidence implicating the involvement of the BM in dengue virus infection. Excruciating bone pain can be a common symptom in dengue patients; hence the term “break-bone fever” was coined and has become synonymous with dengue fever [1]. Pain localized to the BM suggests the involvement of this organ during dengue virus infection. In vitro studies have found that cells in the BM are highly permissive for dengue virus infection [2] and are more so than those from the spleen, lymph node, and thymus [3]. This view is supported by the documentation of a case which reported the transmission of dengue virus from a donor to a recipient as a result of a BM transfusion [4]. In this case study, the donor was at an early stage of infection and did not have any signs of illness. But fever was noticed 2 days after the donation, and it was later confirmed that the donor was indeed infected with dengue type 4 by serological tests.

Bone marrow suppression has long been recognized as a clinical feature contributing to dengue disease. An early investigation of cases in Thailand and Malaysia revealed that the bone marrow mass is at its nadir at the onset of fever and at its peak 2–3 days later (the time when most patients start enrolling in the hospital) [5]–[7]. These kinetics of bone marrow changes makes it very difficult to study this subject in detail for obvious practical reasons [8]. This difficulty is compounded by the bleeding tendencies of these patients making it clinically impractical to acquire BM samples. Thus, except for some earlier investigations of bone marrow during acute infection of dengue patients, the practice of bone marrow sampling is now clinically contra-indicated making it difficult to ascertain the relationship between dengue virus infection and the role of the bone marrow during acute infection. It is important to note that despite decades of research, the primary permissive target cell lineage for dengue virus replication in vivo continues to remain unclear. The fact that acute dengue disease is accompanied with a marked disappearance of megakaryocytes and the stagnation of erythropoiesis [5] in conjunction with thrombocytopenia (a hallmark feature of dengue disease) led us to postulate that dengue virus may indeed target the megakaryocytic lineage.

Recently, dengue virus-induced loss in BM mass was substantiated in the dengue virus coagulopathy model in rhesus macaques [9]. In these animals, the cells capable of generating infectious dengue virus displayed integrin CD61, a cell surface marker specifically expressed by platelets and their megakaryocyte precursors. In order to further understand the nature of dengue virus infection, ex vivo experiments were performed with BM samples from healthy rhesus macaques and humans. The results of these studies showed that i) human BM cells were more permissive than those from rhesus monkeys for dengue virus infection in vitro, as determined by viral RNA and NS-1 quantification assays, ii) densely packed dengue virus-like particles were visualized predominantly in the cytoplasm of multi-lobulated cells, as indicated by electron microscopy (EM), iii) the virus from human and monkey whole BMs were infectious, iv) dengue virus antigen was present in multi-lobulated cells expressing CD61 as determined using immunohistochemical techniques, and v) virus containing cellular debris were engulfed by phagocytic cells, evidenced by EM and histochemical stainings. Taken together, these data are consistent with the view that the megakaryocytes are likely to serve as the major target of dengue virus infection and replication in the BM. We reason that the mechanisms of platelet dysfunction and thrombocytopenia are in part due to the targeting of megakaryocytes in the BM by dengue virus during acute infection. The marked destruction of these cells accompanied by release of pro-inflammatory cytokines such as but not limited to TNF-alpha, IL-1 and IL-6 normally associated with pain, followed by their rapid reconstitution in the BM and probable exudation into the periphery, along with activation of pro-inflammatory cellular signaling pathways, may account for the extreme deep bone pain during the disease.

## Methods

### Ethics Statement for Healthy Rhesus Monkey and Human Bone Marrow Procurement

BM was aspirated from the iliac crest of healthy rhesus monkeys and supplemented with heparin, then infected with virus ex vivo. BM cellularity was analyzed as previously described [9]. All experimental protocols and procedures were conducted following approval by the Emory Institutional Animal Care and Use Committee (IACUC), and all animals were housed at the Yerkes National Primate Research Center of Emory University and cared for in conformance to the guidelines of the Committee on the Care and Use of Laboratory Animals of the Institute of Laboratory Animal Resources, National Research Council and the Health and Human Services [10]. In addition, Yerkes is a facility fully accredited with the Association for Assessment and Accreditation of Laboratory Animal Care International (AAALAC). Animals were fed species specific monkey chow (Purina) ad libitum, with daily supplementation with fresh fruit, and cared for by the Yerkes Veterinary Division. Animals are monitored at least twice daily for clinical and psychological health and provided social enrichment. Animals which develop clinical conditions that cannot be relieved therapeutically are euthanized using the recommended procedures by the American Veterinary Medical Association.

Healthy human BM samples that would otherwise be discarded were obtained from the Stem Cell Processing Laboratory of the Emory Center for Transfusion and Cellular Therapy. The experiments were conducted following appropriate approval by the Emory IRB (Institutional Ethics Committee) with approval protocol #00046063. All patients gave written informed consent for the study.

### In vitro Infection of the Bone Marrow

Results from a pilot study revealed that whole BM without any further processing was just as permissive as fractionated populations of bone marrow cells for dengue virus infection (Figure S1). Thus, all experiments were subsequently performed with unfractionated bone marrow preparations. The total number of nucleated cells were determined as previously described [9]. Dengue virus, strain 16681 [11], grown in Vero cells, was used to infect the unfractionated bone marrow cells ex vivo at an MOI of 0.1. The infected cells following incubation for 2 hours at 37°C were washed X3 with media to remove unbound virus. The infected cells were then re-suspended in 2 ml of culture media and incubated in suspension without shaking and 400 µl of the cell suspension was removed at different time points as indicated in the text. BM smears were prepared by pelleting cells at low speed and applying them to slides.

### Infectious Virus Analysis of BM Supernatant in Vero Cells

Monkey BM cells were similarly infected with an MOI of 0.1 and the supernatant fluids collected on days 2 and 5 following infection. These supernatant fluids were added to Vero cells at 80% confluency. Subsequently supernatant fluids from these Vero cells were collected at the indicated time points and immediately stored at −80°C until real time (RT)-PCR analysis.

Focus forming unit assays were performed by infecting a monolayer of Vero cells in 96-well plates with serial dilutions of supernatant fluids in MEM media collected from human bone marrow cultures. After a two-hour absorption, the cells were overlayed with 1% methylcellulose in EMEM (with 2 mM L-Glutamine, 1 mM sodium pyruvate, 2% FBS, HEPES). Cells were incubated for 3 days and fixed with 3.7% paraformaldehyde. Cells were permeabilized with 1% triton-X for 10 minutes. Cells were washed 5 times with PBS and incubated for 1 hour at 37°C with a predetermined optimum concentration of the monoclonal antibody clone 4G2. Cells were washed 3 times and then incubated with HRP-conjugated rabbit anti-mouse IgG (Dako) for 1 hour at 37°C. Cells were washed 3 times and incubated with diaminobenzidine for 10 minutes.

### FACS Analysis of Bone Marrow Aspirated from DV Infected Rhesus Monkey

Rhesus monkeys (Macaca mulatta) of Indian origin that were part of two separate experiments as previously described [12] were the source of the samples described herein. At different time points post infection, bone marrow was aspirated from the iliac crest in media supplemented with heparin. BM cells were first stained with specific cell surface markers, permeabilized, washed and then incubated with appropriately fluorochrome conjugated monoclonal antibody to dengue viral antigen (clone 3H5), washed and resuspended in FACS buffer and subjected to FACS analysis. All experimental protocols and procedures were conducted following approval by the Emory Institutional Animal Care and Use Committee (IACUC), and all animals were housed at the Yerkes National Primate Research Center of Emory University and cared for in conformance to the guidelines of the Committee on the Care and Use of Laboratory Animals of the Institute of Laboratory Animal Resources, National Research Council and the Health and Human Services [10].

### Periodic Acid Schiff and Giemsa Staining

Staining of cell smears was performed using the Periodic Acid Schiff stain with a PAS kit and Giemsa staining according to the manufacturer’s suggested protocol (Polysciences, Inc., Warrington, PA).

### Immunohistochemistry/immunofluorescent Staining

Immunohistochemical staining for the detection of dengue viral antigen in BM smears was performed by employing the Vectastain ABC immunohistochemistry kit (Vector Laboratories, Inc., Burlingame, CA) according to the manufacturer’s instructions. Mouse anti-E monoclonal antibody (clone 4G2) or isotype-matched control (IgG2a) antibody was utilized in most immunohistochemical staining experiments. However polyclonal anti NS-1 was used in one cell staining with human BM. The stained samples were incubated with 3-amino-9-ethylcarbazole (AEC) or diaminobenzidine (DAB) as an enzyme substrate for peroxidase followed by mounting with DAPI (Invitrogen) or counterstaining with hematoxylin.

The identification of the dengue virus cell lineage consisted of staining of the preparation for dengue viral antigen in addition to a variety of cell lineage specific cell surface markers. Thus appropriate BM smears were fixed onto slides with 4% paraformaldehyde for 20 min and permeabilized with 0.2% triton X-100 for 10 min at RT. The samples were then treated with 0.6% H_2_O_2_ for 30 min to block endogenous peroxidase followed by 30-min incubation with 10% human AB serum. After two washes with PBS, the samples were blocked according to the manufacturer’s instructions and then incubated with mouse anti-E monoclonal antibody (clone 4G2) or its isotype-matched control (IgG2a) antibody at 4°C overnight. The samples were washed three times with PBS and incubated with biotinylated horse anti-mouse immunoglobulin at RT for 30 min followed by three washes with PBS. The samples were then incubated for 30 min each with Vectastain ABC reagent and AEC substrate (all reagents from Vector Laboratories, Inc., Burlingame, CA) for the development of peroxidase signal. Thereafter, the samples were washed three times with PBS, incubated with 10% normal mouse serum for 30 min, and then incubated with FITC-conjugated mouse anti-human CD41a (for megakaryocyte and platelets) or BDCA2 (for phagocytic dendritic cells) antibodies (Genway Biotec, San Diego, CA) for 1 hr. Following washing with PBS, the samples were incubated with 1∶250 dilution of rabbit anti-FITC antibody conjugated to alkaline phosphatase (Sigma Aldrich, St. Louis, MO) and the signal was developed by using Vector blue alkaline phosphatase substrate kit III in the presence of levamisole solution (Vector), an inhibitor of endogenous alkaline phosphatases. The resulting images were captured using a Zeiss microscope equipped with an Axis 5 digital camera. The number of CD41a+DV+, CD41a-DV+, BDCA+DV+, BDCA-DV+ cells were counted by assessing the number of cell surface positive or negative cells among all DV+ cells in 3–5 slides, or number of slides needed to assess 200 4G2 positive cells. Numbers are expressed as a percentage of total DV2+ cells.

For immunofluorescent staining, smears of BM cells on glass slides were fixed with methanol for 5 minutes and rinsed with PBS. The slides were then incubated with 10% human AB serum in PBS at room temperature (RT) for 15 min followed by either mouse anti-NS1 monoclonal antibody (ab41616, Abcam, Cambridge, MA) or isotype-matched control (IgG1) antibody for 1 hr. The slides were washed and then incubated with PE-conjugated goat anti-mouse IgG antibody (eBioscience, San Diego, CA) at a dilution of 1∶1000 for 1 hr. The slides were washed in PBS and then incubated with 10% normal mouse serum in PBS for 30 min at RT to block the remaining binding sites followed by the addition of FITC-conjugated mouse anti-CD61 antibody (eBioscience) for 1 hr. The slides were washed three times with PBS and mounted with DAPI mount reagent (Invitrogen, Carlsbad, CA), and images were captured using a Zeiss microscope equipped with an Axis 5 digital camera.

### Electron Microscopy

BM cells collected at different time intervals following infection were fixed with 2.5% glutaraldehyde in 0.1 M phosphate buffer overnight and then processed for electron microscopy by the Robert P. Apkarian Integrated Electron microscopy Core Facility Service at Emory University.

### Quantitative Real-time RT-PCR (qRT-PCR) for the Detection of Viral RNA

RNA was extracted from 140 µl of culture supernatant fluid isolated from the BM using QIAmp Viral RNA mini kit (QIAGEN). The resultant RNA was then subjected to quantitative RT-PCR using the Taqman RT kit (Perkin Elmer Applied Biosystem) and a Bio-Rad iCycler system according to a previously described method [12]. An aliquot of RNA from a viral stock of DENV was used as a control. The detection limit of this assay was about 100 copies of viral RNA genome equivalents per ml.

### Measurement of NS1 Concentration in Supernatant of Infected Bone Marrow

Supernatant fluids of the culture were collected at the indicated days and stored at −80°C until assay for NS1 as a surrogate measure of virus replication. Standard ELISA was set up to quantify the level of NS1 antigen in the collected supernatant fluid by using purified NS1 antigen (CTK Biotech. Inc, San Diego, CA) to derive a standard curve. Supernatants and various concentrations of NS-1 were incubated with coating buffer on ELISA plates (Nunc Maxisorp) overnight at 4°C. After 2 washes with PBS, samples were blocked with 5% milk in PBS-Tween 20 for 30 minutes at RT. Polyclonal rabbit anti NS-1 antibody (2 µg/ml) in 5% milk was incubated for 1 hour at 37°C. Plates were washed and incubated with horse radish peroxidase-conjugated donkey anti rabbit IgG (1∶2500) in 5% milk for 1 hour at 37°C. Tetramethylbenzidine OptEIA substrate (BD) was prepared and 50 µl was dispensed into individual wells of the microtiter plates and incubated for 5 minutes. The samples were neutralized with 25 µl 4N H_2_SO_4_ and read at OD 490. Time point zero or supernatant fluids from mock infected cells similarly assayed was used to subtract out background signal. Values obtained with the NS1 standard were plotted and used to calculate the amount in the experimental sample.

### Colony Forming Unit Assay

Methylcellulose cultures of the bone marrow cells were used to study the capacity of these cells to produce colonies of hematopoietic origin after dengue virus infection. All necessary reagents were purchased from Stem Cells Technologies, Inc. (Vancouver, Canada), including methylcellulose medium and pre-screened FCS. A total of 1×10^5^ cells were plated in individual 35-mm Petri dishes (Costar, USA) in 1.5 ml of methylcellulose medium with 20% FCS. To promote growth of colony-forming units (CFU), 10 ng/ml SCF, 50 U/ml IL-3, 25 U/ml IL-6, and 2 U/ml erythropoietin were added to detect burst-forming units (BFU)-Erythroid, CFU-Granulocyte-myeloid (CFU-GM) and CFU-megakaryocytes (CFU-MEG). After an incubation period of 12 days at 37°C, *5%* CO_2_, colonies were scored using an inverted microscope. Colonies from such culture dishes were picked for expansion and aliquots subjected to phenotype analysis and pooled for virus infection.

### Treatment with Aldehyde Dehydrogenase (ALDH) Inhibitor

Diethylaminobenzaldehyde (DEAB) was used to treat unfractionated bone marrow cells at 1 mmol/l for 2 days prior to dengue virus infection or immediately after the infection. Untreated and DEAB treated cells that had been infected with dengue virus served as controls. The characteristics of DEAB pre-treated cells were examined before performing dengue virus infection. The infected cells that were DEAB pre-treated, concurrently-treated (added after virus adsorption) and untreated cells were harvested at different time points post infection and subjected to quantitative RT-PCR to determine the levels of viral RNA.

### Statistical Analysis

Statistical analyses were performed with GraphPad Prism V5.04, a GraphPad Software Inc. product. Results were considered statistically significant when p was <0.05.

## Results

### Kinetics of in vitro Viral Replication in Bone Marrow Cells

**Figure 1:**
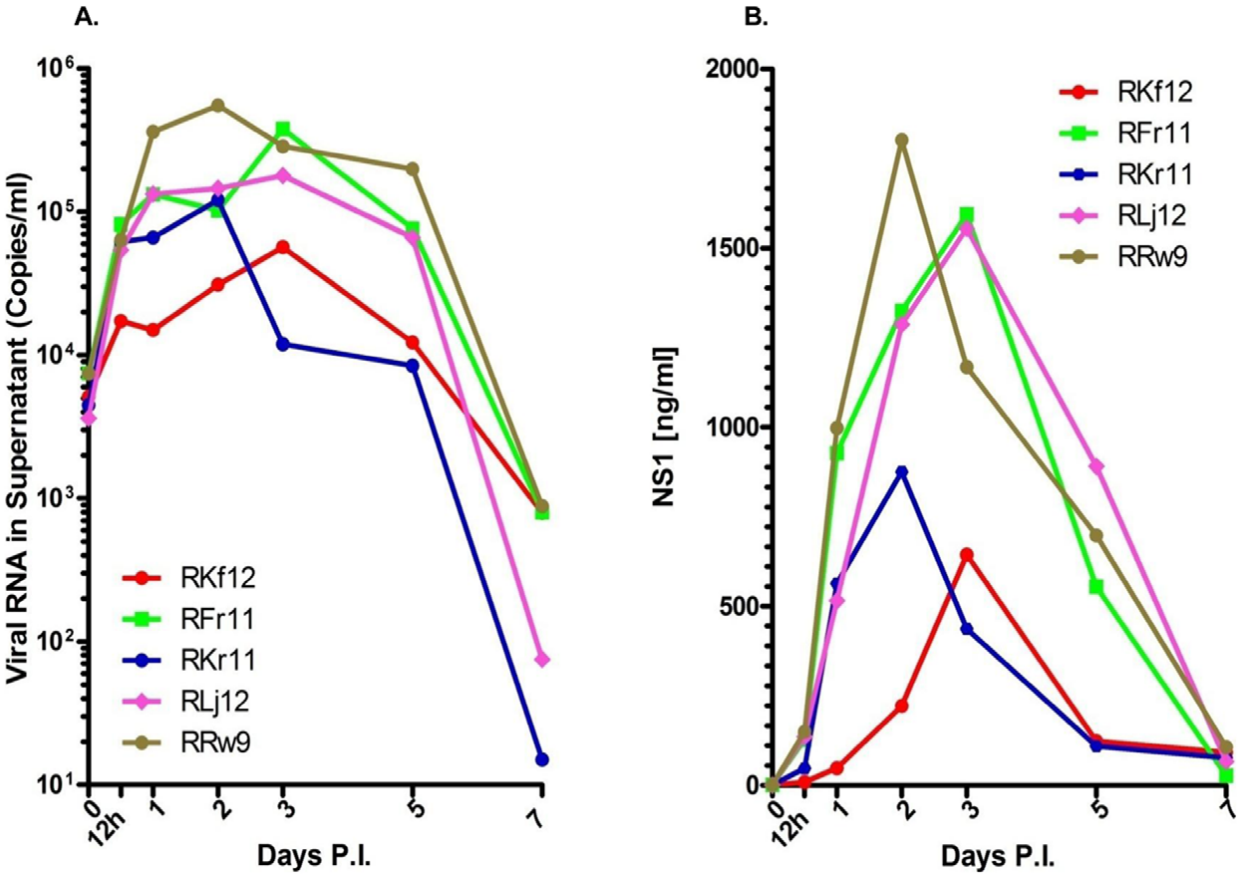
Bone marrow cells from rhesus monkeys are permissive for dengue virus infection in vitro. Fresh whole BM cells were infected with dengue virus at an MOI = 0.1. Supernatant fluids were collected at the indicated times and analyzed by qRT-PCR and nonstructural protein 1 (NS1) ELISA as described in Methods. (A) Viral RNA in supernatants. (B) NS1 in supernatants. Varying degrees of susceptibility to dengue virus infection was noticed.

**Figure 2:**
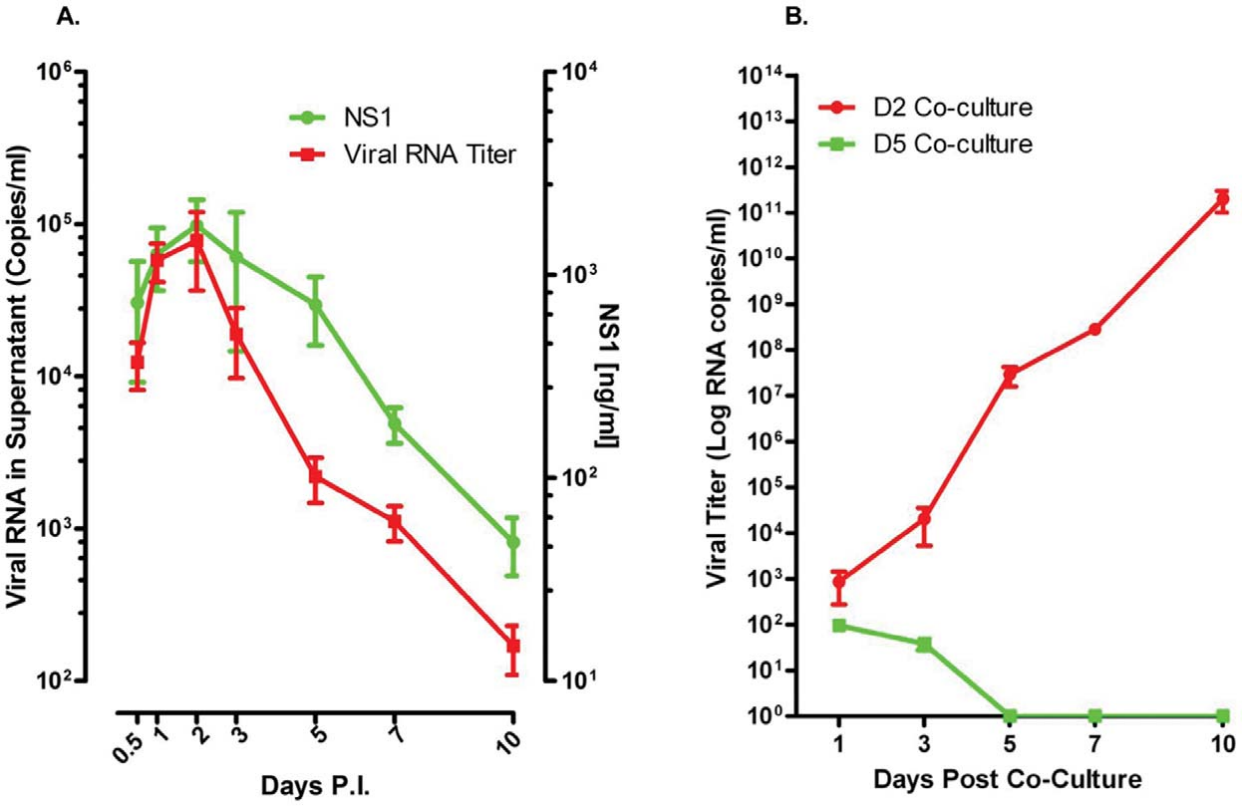
Supernatant fluids early post infection contain infectious virus. All in all, 15 BM samples from healthy rhesus monkeys were studied as described in Figure 1. (A) Kinetics of viral replication in 15 bone marrow cell cultures. Red line indicates RNA titers and green line indicates NS1 protein levels. (B) Infectious virus recovery from supernatant fluids. Supernatants from days 2 (red line) and 5 (green line) were cultured with Vero cells. Approximately equal amounts of viral RNA from 5 randomly chosen monkey BM supernatant fluids were utilized in these culture experiments. Supernatant from day 2 contained infectious dengue virus.

Results from an initial attempt to infect isolated mononuclear cell subsets from the BM of healthy rhesus monkeys indicated that cells optimally permissive for dengue virus infection were in fact present in unfractionated BM (Figure S1). Consequently, all subsequent experiments were performed utilizing unfractionated BM cells to demonstrate the infectability of cells by dengue virus. Studies of the kinetics of virus replication in cultures of ex vivo infected unfractionated BM cell preparations from healthy monkeys showed that whereas these cells were highly permissive for infection by dengue virus, the degree of permissiveness varied with different individual samples (Figure 1A). The levels of nonstructural protein 1 (NS1), a protein that should be expressed by all productively infected cells and a surrogate marker for dengue virus replication, also showed a similar trend (Figure 1B). Viral titers in these BM cultures peaked either on days 2 or 3 after the initiation of infection (Figure 1). As a whole, the trend of viral replication and levels of NS1 in cultures of BM cells from a total of 20 different monkeys was very similar (Figure 2A). However, an increase in the levels of viral RNA does not equate to the production of infectious viral particles. Thus, to demonstrate the infectiousness of the virus obtained in supernatants from infected BM cell cultures, aliquots of randomly selected samples of the cultures from day 2 and 5 containing similar amounts of viral RNA were incubated with fresh Vero cells. Results indicated that virus recovered during the early phase of BM infection contained low but readily detectable levels of infectious virus (Figure 2B). The level of infectious virus in the BM cultures rapidly declined, consistent with an earlier report indicating that supernatants taken from cord blood mononuclear cells at day 8 and co-cultured with C6/36 cells are rarely positive for virus [13].

### Infectability of Megakaryocytes

**Figure 3:**
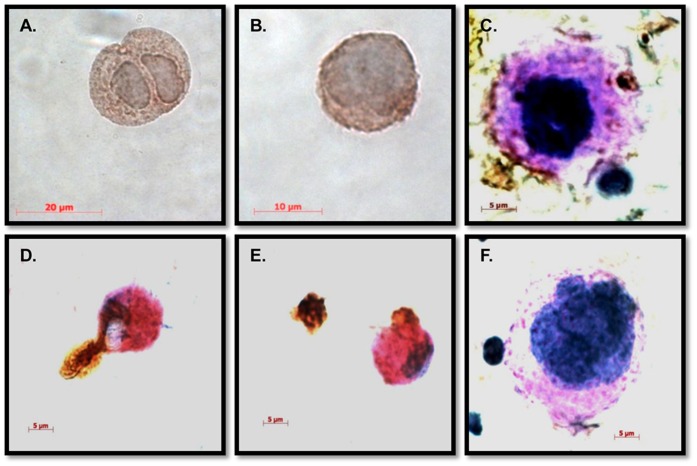
Megakaryocytes were likely the dominant dengue virus antigen positive cells in monkey bone marrow. Smears of bone marrow cells were prepared and immunohistochemical stainings were performed as described in Methods. Dengue antigen (4G2 positivity) is indicated by DAB staining (brown) (A) Dengue viral antigen in a diploid megakaryocyte. (B) Dengue antigen in a multi-lobulated megakaryocyte. (C), Dengue antigen in cellular debris. Red, PAS staining. Blue, hematoxylin staining. (D and E) Dengue viral antigen-containing vesicles engulfed by phagocytic cells. (F) Isotype control.

In an attempt to identify the lineage of BM cells that are permissive for dengue virus infection, BM cultured cells were harvested at different days after infection and smeared onto slides and stained with antibody to dengue viral antigen. Among the cells positive for dengue antigen, those with megakaryocytic characteristics, such as multiple nuclei, were specifically positive for dengue viral antigen at various days p.i. (Figure 3A, 3B, 3C, and Figure S2A, S2B, and S2C), while slides stained with the isotype control (Figure 3F and Figure S2D) were negative. The lineage of DV positive cells was also tested using dual staining for CD41a (a marker of platelets and megakaryocytes) or BDCA2 and DV (Table 1). While CD41a^−/^DV^+^ negative cells were detected at day 1, these cells rapidly declined to undetectable levels, while CD41a^+^DV^+^ cells increased up to day 5 p.i. and stayed above 50% for the duration of the cultures. BDCA2^+^/DV^+^ cells were initially negative and showed a gradual and continuous increase throughout the culture (Figure S3). In addition, dengue viral antigen positive vesicles shedding from apparent megakaryocytic cells were consistently observed (Figure 3C) and phagocytic cells engulfing dengue antigen-positive vesicles could also be detected (Figure 3D and 3E). We interpret these results as suggestive of the megakaryocytic cell lineage as the predominant early target and the bone marrow derived phagocytic cells as critical for subsequent clearance of virus.

**Table 1:** Quantification of monkey bone marrow cells positive for dengue viral antigen^a^.

Days P.I.	1	3	5	7	10
CD41a^+^DV^+^	11.3±2.3	43.4±3.6	50.7±2.9	59.2±7.0	61.4±6.5
CD41a^−^DV^+^	17.5±19	13.6±2.2	10.0±2.4	0.0±0.0	0.0±0.0
BDCA2^+^DV^+^	2.0±0.4	2.6±1.2	41.8±3.6	64.5±8.3	85.5±3.3
BDCA2^−^DV^+^	15.6±2.4	12.2±3.1	4.4±1.5	0.0±0.0	0.0±0.0

a values represent the percentage of surface marker positive or negative among 200 dengue positive (4G2+) cells with 3–5 histochemical stainings.

± standard deviation, P.I., post-infection, BDCA2, plasmacytoid dendritic cell antigen 2.

**Figure 4:**
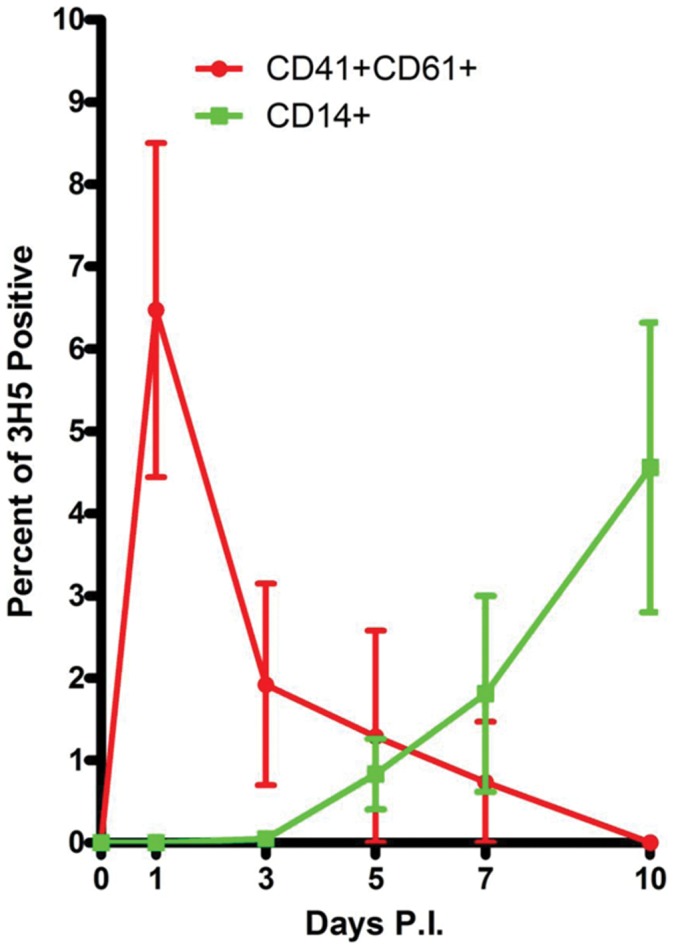
CD61+ cells were the early cells infected by dengue virus bone marrow. Freshly aspirated bone marrows at various time points from DV infected rhesus monkeys were stained with dengue viral specific monoclonal antibody (clone 3H5) and cell lineage markers CD41, CD61, and CD14, and subjected to FACS analysis. Results revealed that viral antigen was observed early in CD61+ cells with a decreasing trend while the opposite trend was evident in CD14+ monocytic cells.

Finally, a kinetic study was performed on BM aspirated from DV infected rhesus monkeys collected at various time points after infection and stained for CD41, CD61, CD14 and DV antigen (Figure 4). Of interest was the finding that whereby viral antigen was observed within CD61+ cells at the early time points post culture followed by a decreasing trend, the opposite trend was evident in CD14+ monocytic cells consistent with our hypothesis stated above.

### Infection of Human Bone Marrow Cells

**Figure 5:**
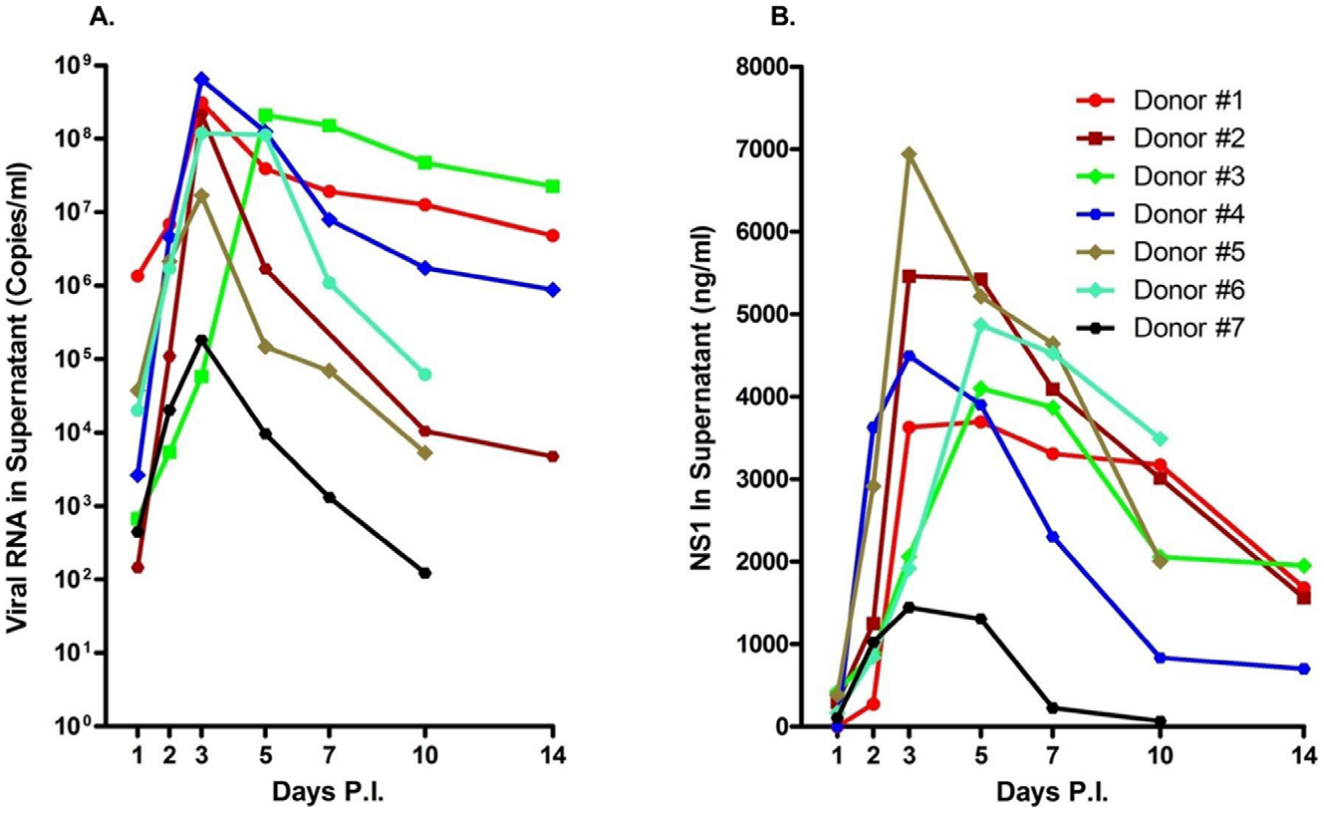
Human bone marrow is permissive for dengue virus infection in vitro. Healthy human BM cells were obtained from the BM transplantation center at Emory University and infected with dengue virus as described in the Methods. Supernatant fluids were collected at the indicated times; viral RNA and NS1 were quantified as described in the Methods. (A) Viral RNA in supernatant fluids. (B) NS1 in supernatant fluids.

**Figure 6:**
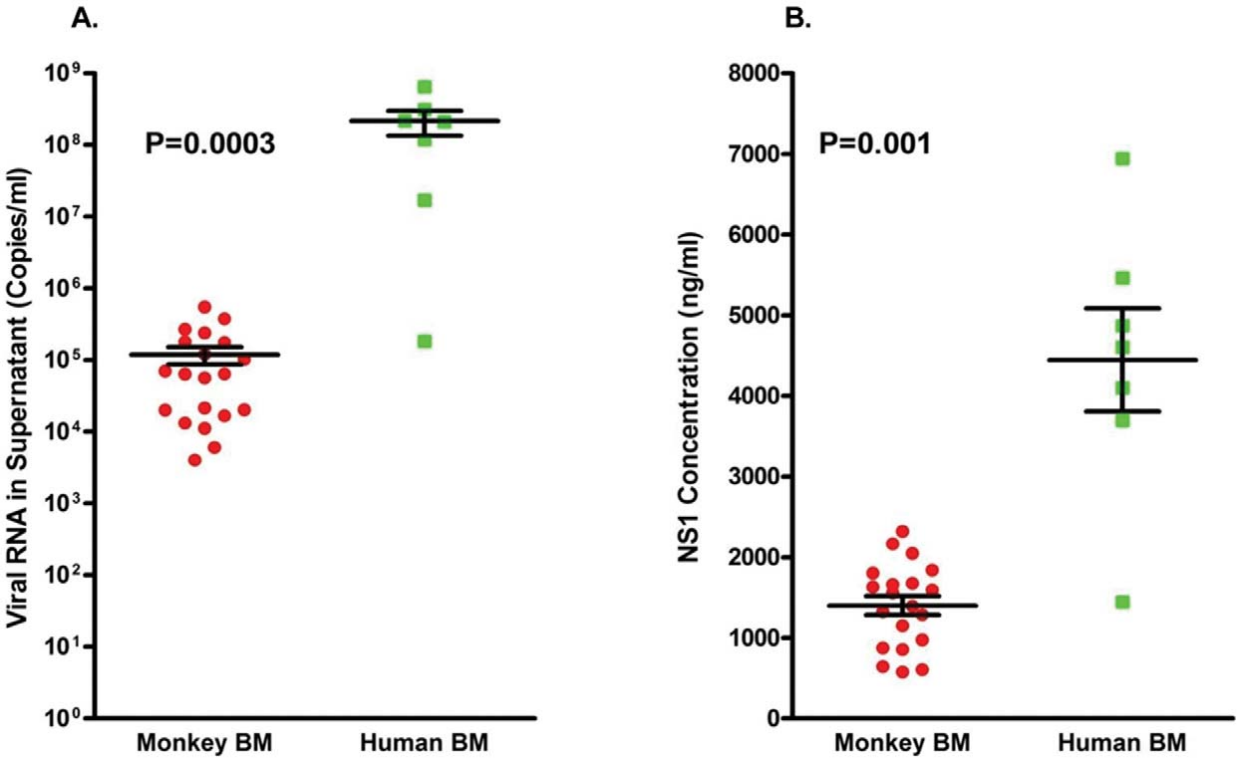
Human bone marrow is more permissive than rhesus macaque bone marrow to dengue virus infection in vitro. (A) A comparison of peak virus genome copy number levels in human and monkey BM cultures. (B) Comparison of NS1 in the supernatant fluid of human and monkey BMs. The levels of viral RNA and NS1 in the supernatant fluid from infected human BM were significantly higher than that from the rhesus monkey.

**Figure 7:**
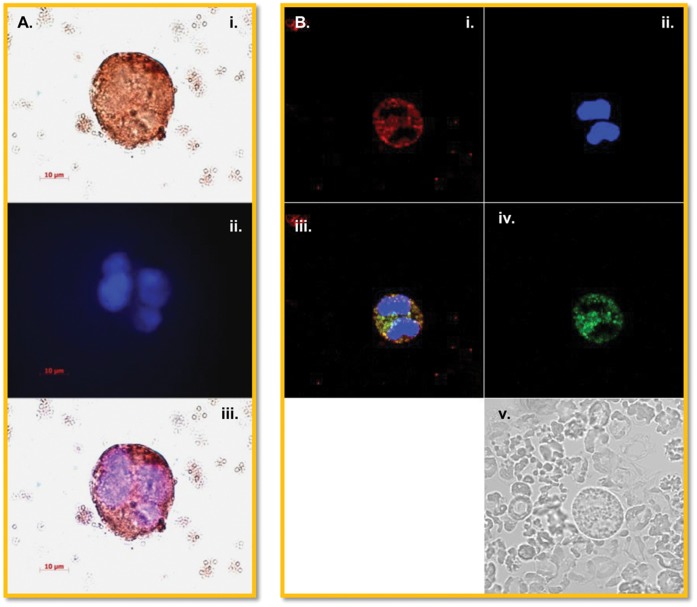
Megakaryocytes from human bone marrow contain dengue virus antigen. Bone marrow smears were prepared and fluorescent cell stainings were performed as described in the Methods. (A) Dengue viral E antigen (identified by 4G2) in tetraploid megakaryocyte in the process of shedding vesicles as evidenced by immunohistochemical staining in the presence of DAPI. Dengue viral antigen (red) and nucleus (blue) (B) Dengue NS1 antigen in a CD61+ megakaryocytic cell depicted by immunofluorescence staining. NS1 (green), CD61 (red) and nucleus (blue). Scale bar,10 µm.

It has been known for a long time that monkeys can be infected by dengue virus, but their levels of viremia are lower than that of human beings. Thus, it was reasoned that studies similar to the above studies should be attempted using human-derived BM cells to derive comparative data. Leftover healthy human BM samples were thus obtained from the BM transfusion center at Emory University School of Medicine and infected with dengue virus in vitro. To our surprise, not only were human BM cells easier to infect with the virus, but, in addition, the levels of virus in the supernatant fluid could reach as high as 10^9^ viral RNA copies per ml, which is similar to the level of viral load in the peripheral blood of dengue patients (Figure 5A). Similar results were also noted in the levels of NS1 in the same supernatants (Figure 5B). Importantly, the pattern of the average focus forming unit (FFU) viral titer was similar but lower than that of the viral RNA titer determined by qRT-PCR assays, peaking on day 3 after infection (Figure S4). The higher viral titers and the detection of NS-1 documented in human BM cultures was statistically significant (Figure 6). BM smears prepared from the human BM cell cultures at different times post-infection were similarly stained with monoclonal antibodies specific to dengue viral antigen and cell surface markers as described above. Results revealed that cells with the megakaryocytic characteristic/marker were positive for dengue viral antigen (Figure 7A and 7B). Viral antigen containing vesicles shedding from a megakaryocyte with a multi-lobulated nucleus were routinely observed (Figure 7A).

### Electron Microscopy Studies

**Figure 8:**
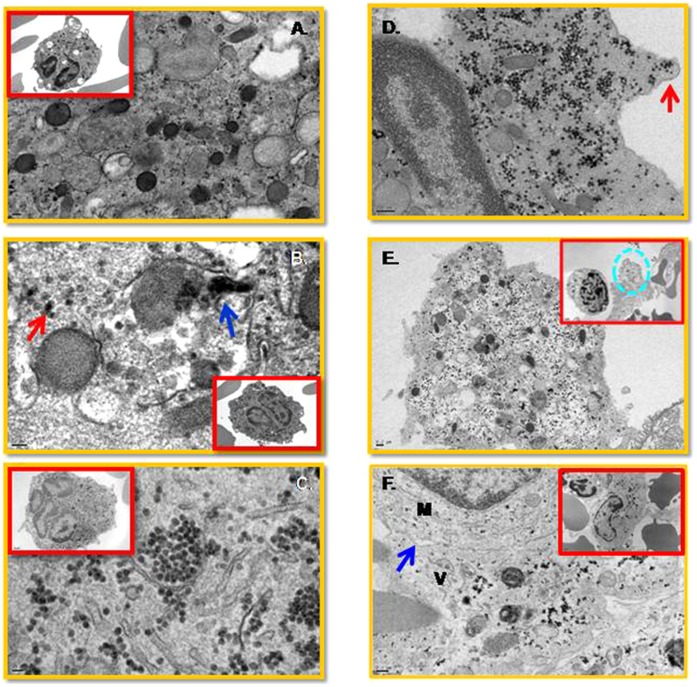
Viral particles are present in megakaryocytes from the human bone marrow. Sample preparations for EM studies were performed as described in the Methods. (A) Uninfected control. (B) Cellular vesicle containing viral particles (single particle, red arrow; cluster of viral particles, blue arrow) inside a diploid megakaryocyte on day one post-infection. (C) Large numbers of viral particles inside the cytoplasm of a multi-lobulated megakaryocyte on day three post-infection. (D) Cytoplasm containing many virus particles shedding off in a vesicle (red arrow). (E) A virion-containing vesicle (dash circle) at the vicinity of an activated mononuclear cell. (F) Virion containing vesicle (V) fusing with a monocyte (M). A zipper junction (blue arrow) is indicated. No viral particles were observed in the monocytes. A scale bar is 0.2 µM.

Electron microscopy (EM) studies were performed on aliquots of bone marrow cell cultures collected on different days after infection. As seen in Figure 8, viral particles appear primarily within multi-lobulated cells (Figure 8), with viral replication complexes visible on day one (Figure 8B) and large numbers of virions present within the cytoplasm by day 3 post-infection (Figure 8C and 8D). As seen, viral particle-containing vesicles appear to be shedding from the cytoplasm (Figure 8D and 8E). We infer that these virus-containing vesicles become engulfed by phagocytic cells at later times post-infection (Figure 8F). EM studies also suggest that phagocytic cells, such as monocytes, are highly activated, featuring numerous vacuoles as early as day one post infection (Figure S5A and S5B). However, virus-like particles were not detectable at this time point in these mononuclear cells (Figure S5C and S5D). In contrast, at later time points post infection, these cells appear to engulf vesicles containing viral particles (Figure S6A and S6B), which seemed to infiltrate the phagocytic cell cytoplasm upon plasma membrane fusion (Figure S6C). The morphology of the viral particles is unclear in these phagocytic cells and are likely degenerated (Figure S6D).

### Colony Forming Unit (CFU) Assays

Numerous heterogeneous progenitor cells are present within the bone marrow; these delicate cells are highly responsive to microenvironment alterations, which likely prompt the differentiation and proliferation of certain cell lineages. Therefore the efficiency of colony formation in the bone marrow post virus infection was evaluated. Data obtained showed that the number of CFUs were reduced post virus infection in a dose-dependent manner (Figure S7). The results of the dose-dependent inhibition are in line with a previous report using purified cord blood mononuclear cells and cord blood CD34^+^ cells [13], [14]. Random colonies were picked, expanded, aliquots identified by Giemsa staining and then the rest infected with dengue virus. Results indicated that cells from the colonies identified as CFU-megakaryocytes were more susceptible to dengue virus infection than colonies identified as CFU-other cells which likely include a mixture of cell lineages. In contrast, cells from CFU-erythrocyte appeared not to support viral replication (Table 2). These results also suggest that hematopoietic stem cells are capable of getting infected with dengue virus. Accordingly, infections were performed with expanded stem cell cultures. Aldehyde dehydrogenase (ALDH) is a receptor on hematopoietic stem cells (HSC) and is a key regulator of HSC differentiation. Human stem cells were treated with the drug DEAB, which interferes with ALDH, down-regulating HSC differentiation and promoting short term stem cell proliferation. After 2 days of treatment with this inhibitor, the majority of cells displayed a multi-lobulated morphology, consistent with the view that this morphology was due to the differentiation of megakaryocytes (Figure S8). These cells were much more permissive to dengue virus infection than untreated or concurrently treated BM cells (Figure S9 and Figure S10A). Immunohistochemical staining revealed that these cells expressed CD41a, indicating they were likely of megakaryocytic origin (Figure S10B and S10C). Viral antigen was observed on globoid-like vesicles that were undergoing budding from the surface of the cells (Figure S10B and S10C).

**Table 2:** Infectivity of dengue virus in Colony Forming Unit cells picked from human bone marrow^a^.

Days P.I.	0^b^	1	3	5	7	10
CFU-other cells	74^c^	215	198	136	124	103
Fold Increase^d^	0	190.5	167.6	83.3	67.6	39.2
CFU-Megakaryocytes	82	114	363	145	92	26
Fold Increase	0	39.5	344.3	77.5	12.1	0
CFU-Erythroid	3530	1000	657	433	194	0
Fold Increase	0	0	0	0	0	0

a Cells were characterized based-upon their morphology and giemsa staining characteristics.

b Day 0 means the time point 2 hours after adsorption, in which culture supernatants were extensively washed for unbound virus. The amount of residual virus in the culture supernatant was determined and used as the baseline.

c Quantification determined by qRT-PCR (unadjusted copy number per 140 µlof the supernatant).

d The fold increase relative to the viral titer at Day 0, supernatant at 2 hours post-infection, was calculated.

## Discussion

It is well known that the bone marrow is composed of a complex and heterogeneous mixture of cell lineages that can vary greatly in composition from individual to individual. This delicate compartment is highly sensitive to any subtle stimulation, which can dramatically change the cellular constituents and its functional capacity. The hierarchical order among the various cell lineages is critical in orchestrating the activities of the host and sustaining homeostasis. The plasticity of hematopoietic progenitor cells in the BM bestows upon them the ultimate power to restore homeostasis. Infectious agents are a type of stimulation that likely disturb the equilibrium, requiring BM progenitors to respond to re-establish order.

Dengue is one of the most important vector-borne diseases in humans. Although the disease predominantly circulates in tropical and subtropical zones, it has recently been acknowledged as a potential public health threat in several other locations around the world. The majority of those infections remain asymptomatic, but many experience dengue fever (DF) that is a self-limited illness. Only a small percentage of affected subjects progress to the very severe and life-threatening clinical form termed dengue hemorrhagic fever (DHF) accompanied with shock syndrome (DSS), which is characterized by increased vascular permeability, plasma leakage and internal bleeding. The degree of thrombocytopenia has been demonstrated to significantly correlate with the severity of the disease. Understanding the mechanisms accounting for the drop in platelet counts has been one of the central themes for several decades. The following processes, acting successively or in combination, have been demonstrated to interfere with the number of platelets in the peripheral blood of dengue patients: reduced platelet production through early transient marrow suppression with damage to megakaryocytes [9], [15]; platelet aggregation with endothelial cells upon dengue virus activation [16], [17]; hemo-phagocytosis [18], [19]; and finally, immune destruction of platelets displaying dengue-antibody complexes on their membranes [20]. Profound hematopoietic suppression has been noted to occur in dengue virus infected patients early post infection occurring prior to hospital admission [5]–[7]. Thus, direct suppressive action of the virus on megakaryocytes was suggested as a mechanism contributing to thrombocytopenia long ago [21], however this hypothesis was never properly evaluated and remained un-confirmed.

We first observed that unfractionated BM cultures are highly permissive for dengue virus infection relative to purified populations of BM mononuclear cells. A requirement for the presence of other cell lineages for optimum growth and survival of megakaryocytes and/or that the Ficoll-Paque gradient separation procedure may serve to shear critical cell surface molecules required for optimal infection and thus account for the difference. Results obtained using a combination of immunohistochemical staining and electron microscopy imaging techniques authenticate that multi-lobulated megakaryocytes are highly permissive for dengue virus infection in vitro. This can be inferred from previous findings indicating that hematopoietic cells other than megakaryocytes are very seldom polyploid in healthy BM [22]–[24]. Productive infection of these megakaryocytic cells likely plays an important part in the development of thrombocytopenia characteristic of dengue infected patients.

Megakaryocytes are one of the most unique cells in the mammalian system, accounting for only 1% of healthy BM. They express all proteins required for cell division and yet never divide to generate daughter cells. The surface area of the cell membrane progressively expands to an enormous size, which then, via internal operational signaling, extends itself into a demarcation membrane that sheds to produce platelets, a mechanism likened to apoptosis. Correspondingly, the contents of the chromosome increase, continuously doubling the genome to numbers as large as 128N. Each megakaryocyte can produce between 3000 to 5000 platelets depending upon the size of the membrane and thus differentiation stage of the cell [25]. Thrombopoeisis normally takes 4 to 7 days for completion with 2/3 of the newly produced platelets destined to the peripheral blood for circulation, while 1/3 becomes sequestered within the spleen. The multi-lobulated cells observed during dengue virus infection appeared to be smaller in size, likely classified as micro-megakaryocyte, as opposed to a late stage megakaryocyte population. This could be an indication that dengue virus infection may inhibit differentiation, transiently delaying and/or inhibiting the doubling of the genome and expansion of the membrane, resulting in a reduced efficiency in platelet production. Furthermore, if platelets are produced from these infected cells, they are likely dysfunctional. Perhaps, this may be one of the reasons why in some patients, the levels of platelet counts are within normal range, but hemorrhagic manifestations are still observed.

Interestingly, despite careful study, we were unable to observe viral particles in activated monocytes of the BM during the early days of infection. However, we frequently observed virus containing vesicles becoming engulfed by monocytes and degenerated virus-like particles in the cell cytoplasm at later times post infection. The evidence is in line with a previous publication, in which the authors report that only cells from the bone marrow are capable of supporting dengue virus replication after a side by side comparison with cells from other monocyte rich organs (spleen, lymph node, and thymus) [26]. The activated mononuclear cells we observed could well be inflammatory monocytes that have the ability to differentiate into dendritic cells equipped with a high degree of phagocytic activity [27]. Interestingly, it has been suggested that the elimination of apoptotic bodies by phagocytic cells is a pathway of dengue virus clearance in infected tissues [28] and that the shedding of platelets is a mechanism operationally similar to apoptosis in megakaryocytes [29]. This may perhaps explain the observation that BDCA2+ cells become antigen-positive late in infection, probably due to phagocytosis of dengue-containing apoptotic debris. Nevertheless, the results are in line with reports on the importance of monocytes/macrophages in the clearance of virus in the circulation [12], [30]–[33].

In addition, results from the DEAB inhibition assays indicated that viral yields in the supernatants were readily detectable in cells with multi-lobulated nuclei. Interestingly, it has been reported that cells highly resistant to gamma irradiation are concentrated in DEAB-treated hematopoietic stem cells and that they are likely to be multi-lobulated megakaryocytes. Importantly, it has already been documented that dengue viral titers are not reduced in bone marrow cells treated with gamma radiation and that antibody-mediated dengue virus infection can occur only in rhesus macaque cells isolated from the bone marrow [3]. Furthermore, megakaryocytes express FcγRIIa and FcγRIIb on their plasma membrane surface [34]–[36]. Thus, upon re-exposure to a heterologous dengue viral strains, antibody mediated entry may occur in bone marrow tissue in vivo through these Fc receptors and increase the occurrence of disease symptoms, such as thrombocytopenia.

Reduced platelet count is a common clinical feature seen not only in dengue patients but also in people infected with other infectious agents. Junin virus, the causative agent of Argentinian hemorrhagic fever, [37], [38], murine lymphoid viruses [39] and HIV [40], [41], the causative agent of AIDS have been documented to attack the megakaryocytes as well. The potential mechanism at the origin of this preference may be that megakaryocytes are defective in interferon alpha/beta synthesis [36], [42], a critical inhibitory molecule that can limit the gene expression of many viruses. Perhaps, with their defective defense machinery, megakaryocytes are an easy target for multiple pathogens.

In conclusion, utilizing a variety of approaches, our results suggest that dengue virus can infect a subset of cells from the bone marrow. These cells are CD61+ and CD41a+ and have characteristics of megakaryocytes. This may partially explain why bone marrow mass is affected and patients suffer excruciating bone pain during the acute stage of infection. This is likely to contribute to thrombocytopenia and explain the commonality of platelet dysfunction. This data suggests the need to evaluate the functionality of the bone marrow cells during dengue virus infection. The targeting of anti-viral immune responses to the bone marrow that has the potential to reduce overall viremia, may pave the way to the development of better vaccine candidates and therapeutic drug treatments.

## Supporting Information

Figure S1 **Whole bone marrow supports dengue virus replication.** Freshly obtained monkey bone marrow was infected with dengue virus at an MOI = 0.1 and supernatants were collected at the indicated times. Viral RNA was quantified as previously described [9]. (A) Increased viral RNA levels in whole bone marrow. A portion of the same whole bone marrow specimen was subjected to Ficoll-Paque gradient fractionation; two fractions, (B) red blood cells (RBC) and (C) bone marrow mononuclear cells (BMMC), were collected and infected with dengue virus. Both fractions did not appear to support dengue virus replication.
(TIF)

Figure S2 **Dengue viral antigen was dominantly observed in multi-nucleated cells.** Immunohistochemical staining was performed as described in the Methods. (A) and (B) Dengue viral antigen (stained with 4G2) was specifically observed in multi-nucleated cells. (C) DV infected cells were stained with DV antibody after lysis of RBCs. (D) Isotype control staining.
(TIF)

Figure S3 **Dengue viral antigen (indicated with 4G2 antibody) is present in CD41a+ cells and not in BDCA2+ cells at early time points of infection.** Monkey bone marrow smears were prepared from whole bone marrow infected with dengue virus at an MOI = 0.1. Cells were harvested at the indicated times, smeared onto slides, and stained with the indicated cell markers, CD41a (Blue), marker for platelets, and BDCA2 (Blue), maker for plasmacytoid dendritic cells, and antibody specific to dengue viral antigen (Red).
(TIF)

Figure S4 **Quantification of infectious viral titers with focus forming unit assays (FFA).** The viral titer and the infectivity of the virus in the collected specimens were determined using a FFA. [12]. Titers were expressed as FFU per ml. The pattern of the average viral titer was similar to that of viral RNA titer determined by qRT-PCR assays, peaking on day 3 after infection.
(TIF)

Figure S5 **Monocytes from infected human bone marrow appear uninfected and activated.** Infected bone marrows were processed for EM investigations as described in methods. (A and B) Activated and vacuole-loaded phagocytic cells, likely monocytes or macrophages. (C and D) Absence of discernible viral particles or replication complexes in vacuolated cytoplasm of activated monocytes or macrophages. The images were captured after one day of infection.
(TIF)

Figure S6 **Phagocytic cell engulfs virion-containing vesicles.** Images were captured by EM of human whole bone marrow on day 5 after infection. (A) A vesicle loaded with virus-like particles (V) fusing with a monocyte or macrophage (M). (B) Zipper junction (circle) at the fusion point. (C) Virions transfering from the vesicle to the cytoplasm of the phagocytic cell. (D) Degenerated viral particles inside the cytoplasm of phagocytic cells on day 7 after infection.
(TIF)

Figure S7 **The efficiency of colony formation in human bone marrow was inhibited by dengue virus in a dose-dependent manner.** Healthy human bone marrow was exposed to dengue virus at an MOI = 1 or 0.1 for two hours. Unbound virus was removed with three washes of media, and cells were cultured with CFU media according to the protocol suggested by the manufacture (StemCells Technologies Inc., Vancouver, Canada). Uninfected human bone marrow was used as control. (A) Fewer and smaller colonies are observed with increased MOI. (B) Quantification of colony formation in the presence and absence of dengue virus. Y-axis indicates the number of colonies per dish. Data was tabulated from three replicates performed on different days. There is a statistically significant inhibition of colony formation in human bone marrows exposed to dengue virus.
(TIF)

Figure S8 **Multi-lobulated cells were the dominant population present in monkey bone marrows treated with the drug diethylaminobenzaldehyde (DEAB).** Bone marrows were treated with DEAB, an inhibitor for aldehyde dehydrogenase (ALDH), at a concentration of 1 µmol/l for two days. Cellular morphology of cells after Wright Giemsa staining was captured with a Zeiss inverted microscope.
(TIF)

Figure S9 **Bone marrow cells pre-treated with DEAB are permissive for dengue virus infection in vitro.** Monkey whole BM cell samples treated in three different ways (DEAB-untreated (green), 2 day pre-treated (red) and DEAB-treated concurrently with the infection (blue) were performed as described in methods. Results from two representative monkeys are shown. The peak in genome titer was at days 2 or 3 post initiation of infection.
(TIF)

Figure S10 **Monkey bone marrows treated with DEAB for two days are highly permissive to dengue virus infection.** (A) RNA quantification was performed with the three experimental groups described in Figure S9 from four monkeys: 2-DEAB, bone marrow pre-treated with DEAB for two days before virus infection; WBM, DEAB-untreated and DENV-infected whole bone marrow; DEAB, DEAB added to culture immediately after dengue virus infection. The kinetic fold max increase in viral titer compared to that at time 0, or two hours after absorption, was calculated. The peak fold increase in viral titers is presented. Cells were cytospun onto slides and immunohistochemical staining for CD41a and dengue E antigen was performed as described in the Methods. (B) IgG2a Isotype control and CD41a. (C) Viral antigen observed in megakaryocyte that was ongoing vesicle-shedding. Dengue E antigen (brown), CD41 (blue) and nucleus (DAPI stained). 1. Noisakran S, Onlamoon N, Hsiao HM, Clark KB, Villinger F, et al. (2012) Infection of bone marrow cells by dengue virus in vivo. Exp Hematol 40: 250–259 e254. 2. Onlamoon N, Noisakran S, Hsiao HM, Duncan A, Villinger F, et al. (2010) Dengue virus-induced hemorrhage in a nonhuman primate model. Blood 115: 1823–1834.
(TIF)
