# Supplementary figures and images for: Multiploid CD61+ Cells Are the Pre-Dominant Cell Lineage Infected during Acute Dengue Virus Infection in Bone Marrow
Source: PLoS One. 2012 Dec 27;7(12):e52902. doi: 10.1371/journal.pone.0052902 (PMC3531377; doi:10.1371/journal.pone.0052902)

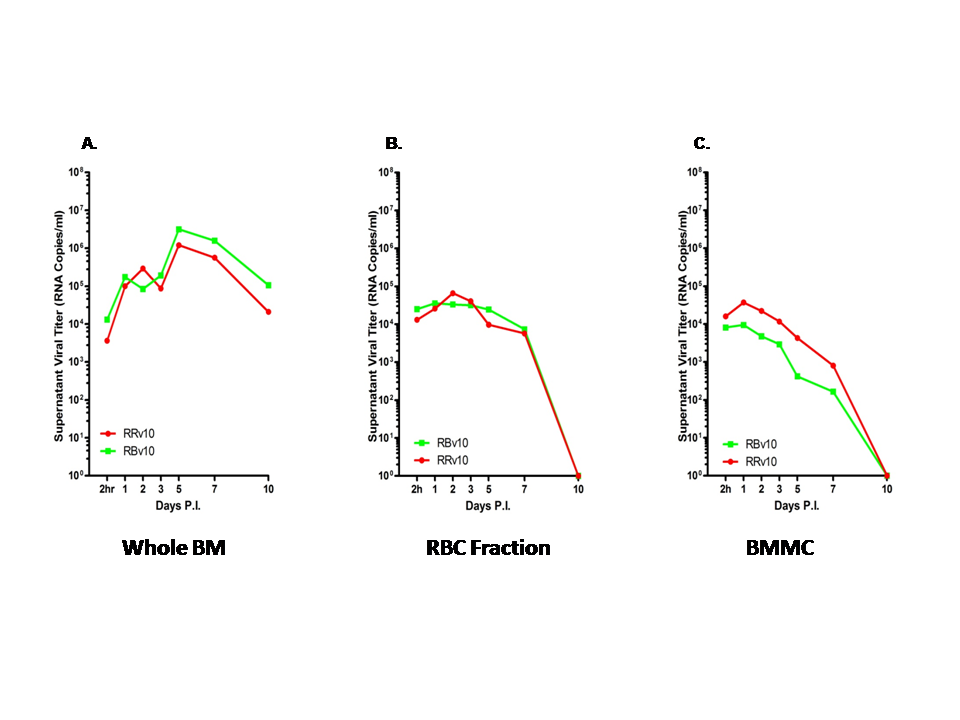

Supplement: Figure S1 — Whole bone marrow supports dengue virus replication. Freshly obtained monkey bone marrow was infected with dengue virus at an MOI = 0.1 and supernatants were collected at the indicated times. Viral RNA was quantified as previously described [9]. (A) Increased viral RNA levels in whole bone marrow. A portion of the same whole bone marrow specimen was subjected to Ficoll-Paque gradient fractionation; two fractions, (B) red blood cells (RBC) and (C) bone marrow mononuclear cells (BMMC), were collected and infected with dengue virus. Both fractions did not appear to support dengue virus replication. (TIF) [file pone.0052902.s001.tif]

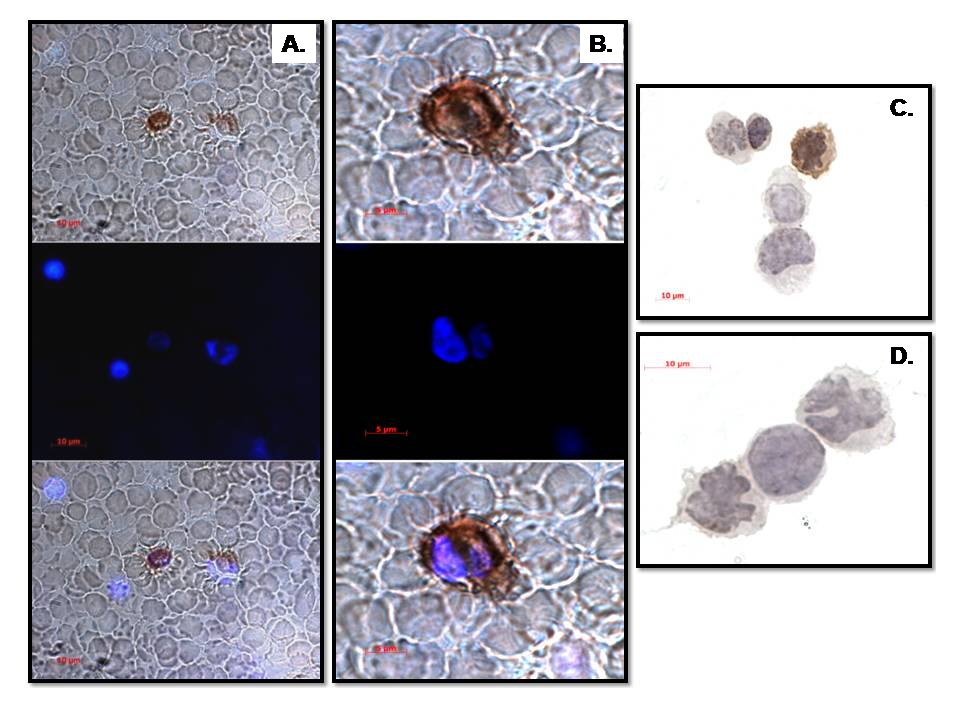

Supplement: Figure S2 — Dengue viral antigen was dominantly observed in multi-nucleated cells. Immunohistochemical staining was performed as described in the Methods. (A) and (B) Dengue viral antigen (stained with 4G2) was specifically observed in multi-nucleated cells. (C) DV infected cells were stained with DV antibody after lysis of RBCs. (D) Isotype control staining. (TIF) [file pone.0052902.s002.tif]

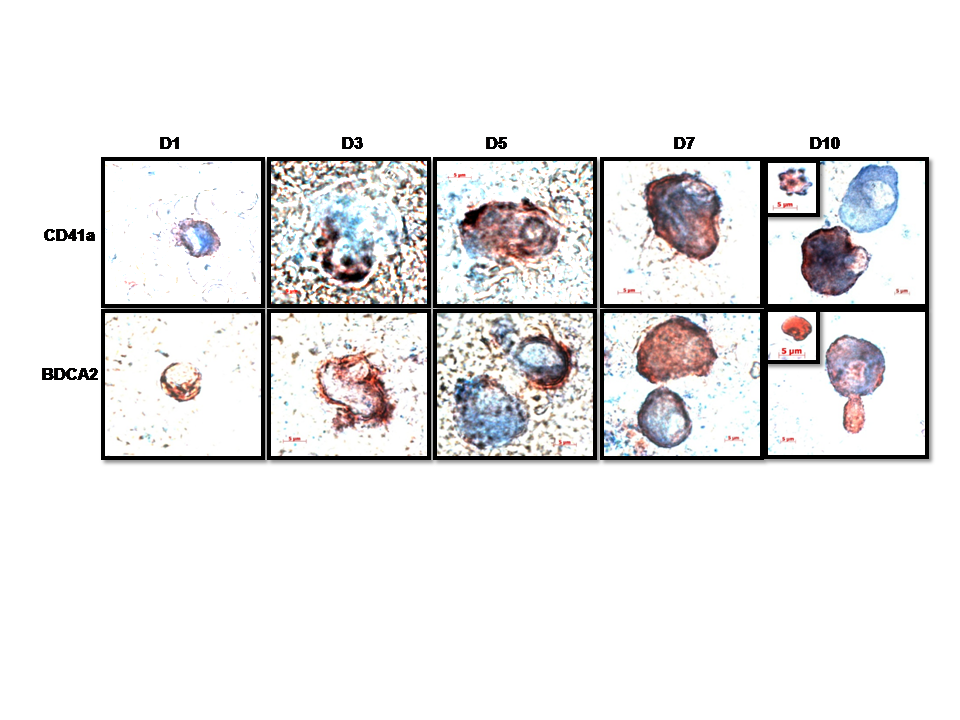

Supplement: Figure S3 — Dengue viral antigen (indicated with 4G2 antibody) is present in CD41a+ cells and not in BDCA2+ cells at early time points of infection. Monkey bone marrow smears were prepared from whole bone marrow infected with dengue virus at an MOI = 0.1. Cells were harvested at the indicated times, smeared onto slides, and stained with the indicated cell markers, CD41a (Blue), marker for platelets, and BDCA2 (Blue), maker for plasmacytoid dendritic cells, and antibody specific to dengue viral antigen (Red). (TIF) [file pone.0052902.s003.tif]

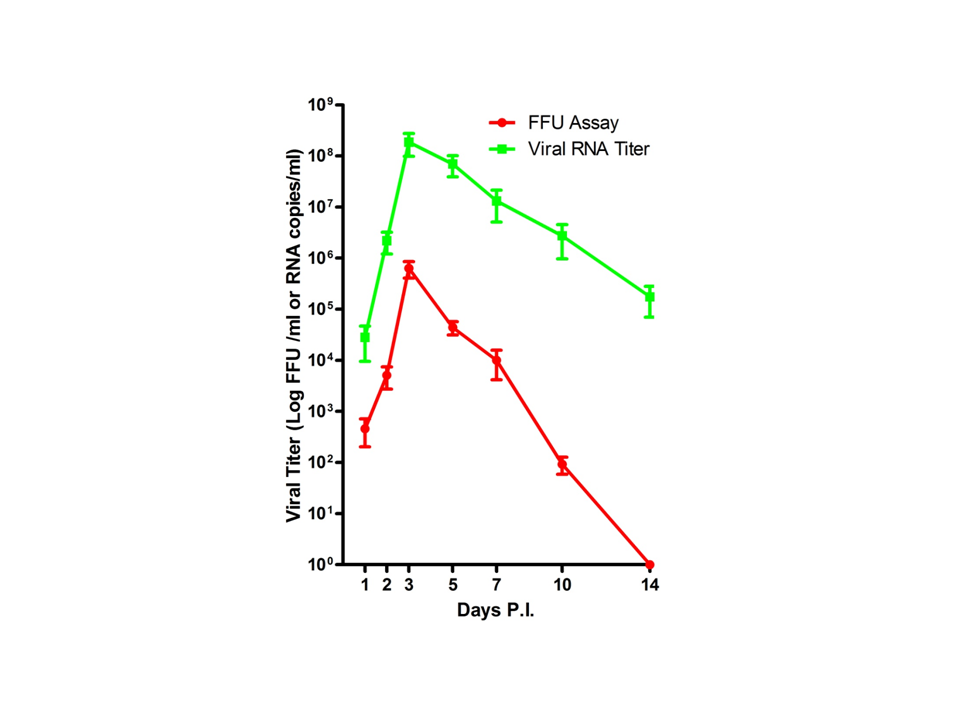

Supplement: Figure S4 — Quantification of infectious viral titers with focus forming unit assays (FFA). The viral titer and the infectivity of the virus in the collected specimens were determined using a FFA. [12]. Titers were expressed as FFU per ml. The pattern of the average viral titer was similar to that of viral RNA titer determined by qRT-PCR assays, peaking on day 3 after infection. (TIF) [file pone.0052902.s004.tif]

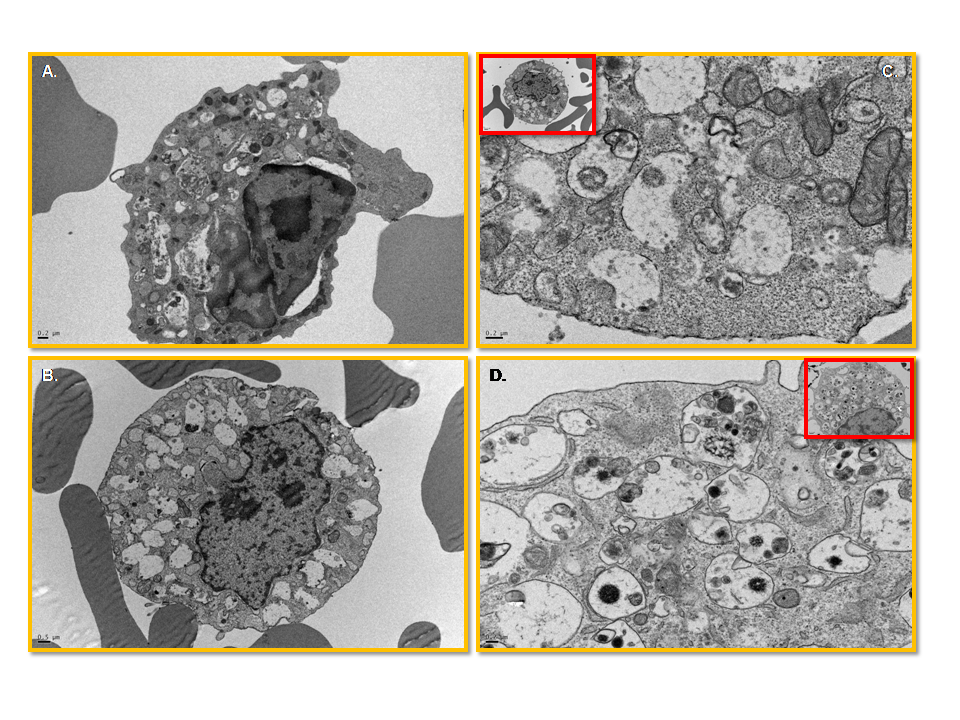

Supplement: Figure S5 — Monocytes from infected human bone marrow appear uninfected and activated. Infected bone marrows were processed for EM investigations as described in methods. (A and B) Activated and vacuole-loaded phagocytic cells, likely monocytes or macrophages. (C and D) Absence of discernible viral particles or replication complexes in vacuolated cytoplasm of activated monocytes or macrophages. The images were captured after one day of infection. (TIF) [file pone.0052902.s005.tif]

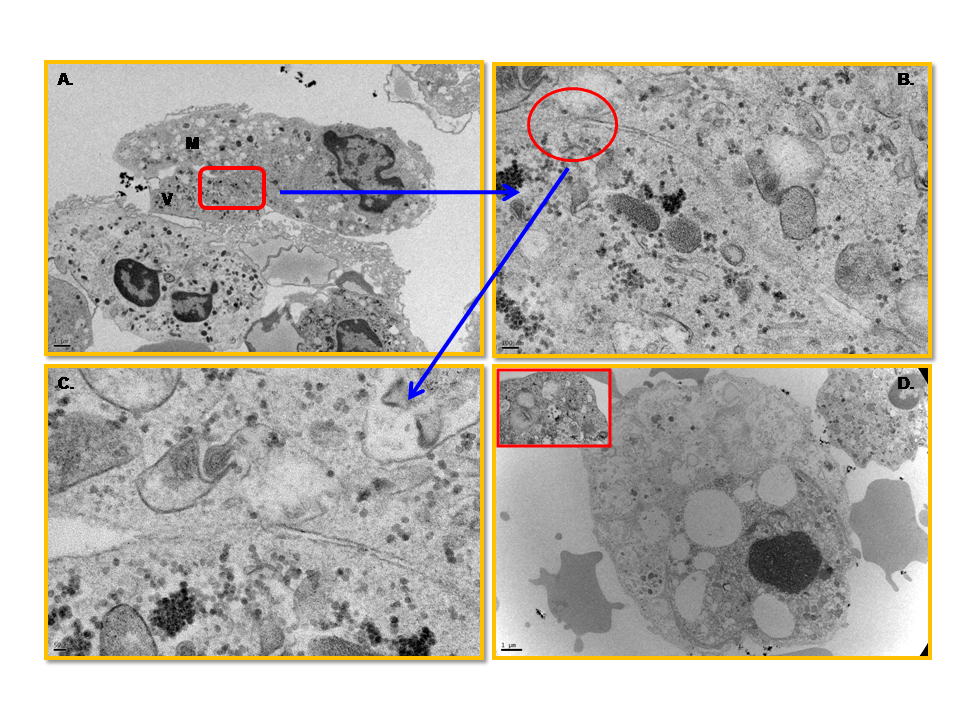

Supplement: Figure S6 — Phagocytic cell engulfs virion-containing vesicles. Images were captured by EM of human whole bone marrow on day 5 after infection. (A) A vesicle loaded with virus-like particles (V) fusing with a monocyte or macrophage (M). (B) Zipper junction (circle) at the fusion point. (C) Virions transfering from the vesicle to the cytoplasm of the phagocytic cell. (D) Degenerated viral particles inside the cytoplasm of phagocytic cells on day 7 after infection. (TIF) [file pone.0052902.s006.tif]

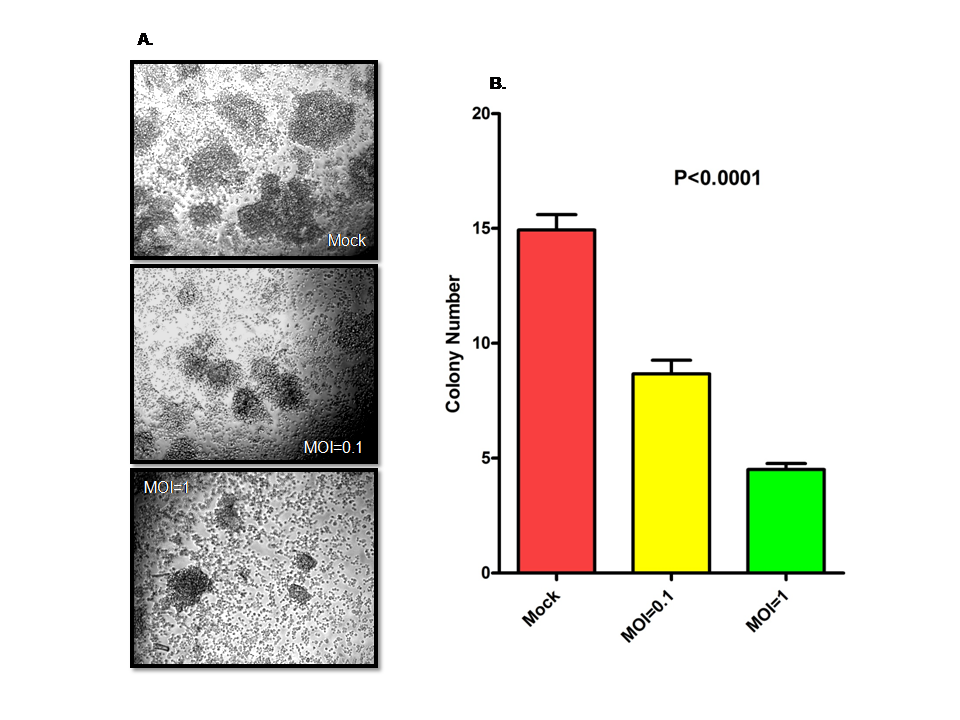

Supplement: Figure S7 — The efficiency of colony formation in human bone marrow was inhibited by dengue virus in a dose-dependent manner. Healthy human bone marrow was exposed to dengue virus at an MOI = 1 or 0.1 for two hours. Unbound virus was removed with three washes of media, and cells were cultured with CFU media according to the protocol suggested by the manufacture (StemCells Technologies Inc., Vancouver, Canada). Uninfected human bone marrow was used as control. (A) Fewer and smaller colonies are observed with increased MOI. (B) Quantification of colony formation in the presence and absence of dengue virus. Y-axis indicates the number of colonies per dish. Data was tabulated from three replicates performed on different days. There is a statistically significant inhibition of colony formation in human bone marrows exposed to dengue virus. (TIF) [file pone.0052902.s007.tif]

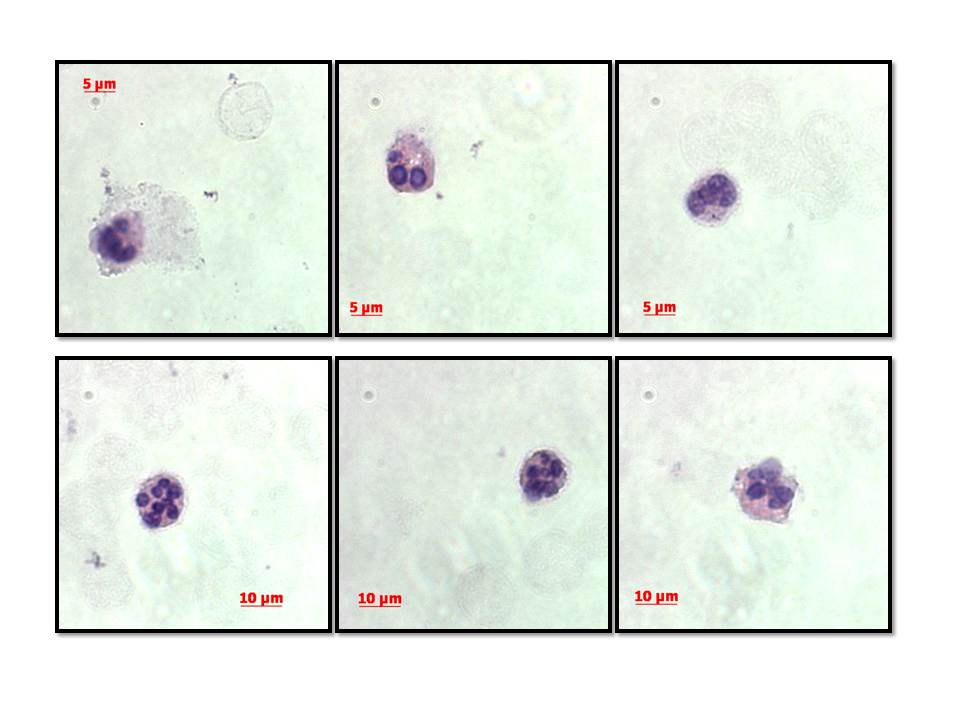

Supplement: Figure S8 — Multi-lobulated cells were the dominant population present in monkey bone marrows treated with the drug diethylaminobenzaldehyde (DEAB). Bone marrows were treated with DEAB, an inhibitor for aldehyde dehydrogenase (ALDH), at a concentration of 1 µmol/l for two days. Cellular morphology of cells after Wright Giemsa staining was captured with a Zeiss inverted microscope. (TIF) [file pone.0052902.s008.tif]

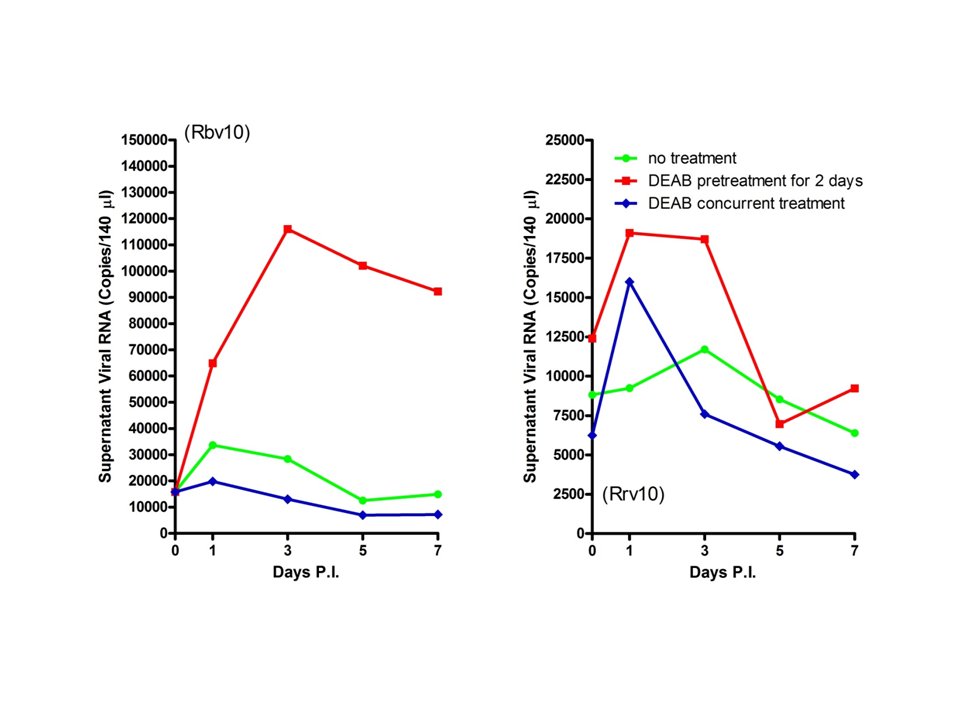

Supplement: Figure S9 — Bone marrow cells pre-treated with DEAB are permissive for dengue virus infection in vitro. Monkey whole BM cell samples treated in three different ways (DEAB-untreated (green), 2 day pre-treated (red) and DEAB-treated concurrently with the infection (blue) were performed as described in methods. Results from two representative monkeys are shown. The peak in genome titer was at days 2 or 3 post initiation of infection. (TIF) [file pone.0052902.s009.tif]

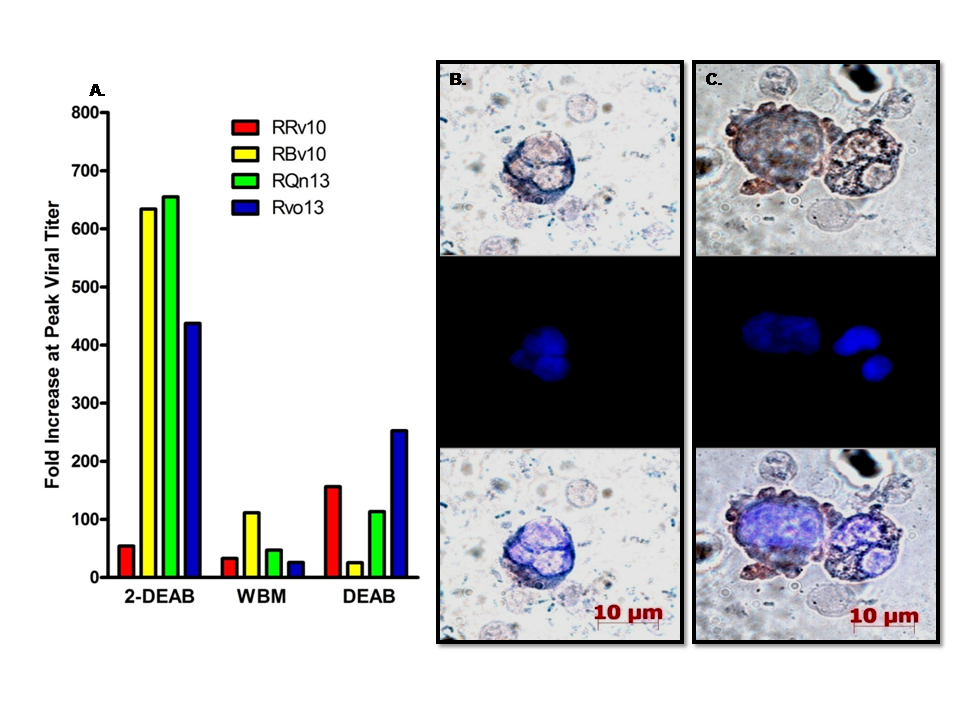

Supplement: Figure S10 — Monkey bone marrows treated with DEAB for two days are highly permissive to dengue virus infection. (A) RNA quantification was performed with the three experimental groups described in Figure S9 from four monkeys: 2-DEAB, bone marrow pre-treated with DEAB for two days before virus infection; WBM, DEAB-untreated and DENV-infected whole bone marrow; DEAB, DEAB added to culture immediately after dengue virus infection. The kinetic fold max increase in viral titer compared to that at time 0, or two hours after absorption, was calculated. The peak fold increase in viral titers is presented. Cells were cytospun onto slides and immunohistochemical staining for CD41a and dengue E antigen was performed as described in the Methods. (B) IgG2a Isotype control and CD41a. (C) Viral antigen observed in megakaryocyte that was ongoing vesicle-shedding. Dengue E antigen (brown), CD41 (blue) and nucleus (DAPI stained). 1. Noisakran S, Onlamoon N, Hsiao HM, Clark KB, Villinger F, et al. (2012) Infection of bone marrow cells by dengue virus in vivo. Exp Hematol 40: 250–259 e254. 2. Onlamoon N, Noisakran S, Hsiao HM, Duncan A, Villinger F, et al. (2010) Dengue virus-induced hemorrhage in a nonhuman primate model. Blood 115: 1823–1834. (TIF) [file pone.0052902.s010.tif]
